# From Risk to Flourishing: Organizational Resources in Seasonal Tourism Work

**DOI:** 10.3390/ijerph23060779

**Published:** 2026-06-10

**Authors:** Stefania Fantinelli, Michela Cortini, Morena Santoriello, Leonardo Pagano, Teresa Galanti

**Affiliations:** 1 CePSI—Research Center in Applied Psychology, Department of Theoretical and Applied Sciences, e-Campus University, 22060 Novedrate, Italy; 2 Department of Psychology, University G. d’Annunzio of Chieti—Pescara, 66100 Chieti, Italy; cortini@unich.it (M.C.); morena.santoriello@phd.unich.it (M.S.); teresa.galanti@unich.it (T.G.); 3 Ministry of Defence, 00187 Rome, Italy; leonardo_pagano.549114@unifg.it

**Keywords:** seasonal workers, positive psychology, mixed-method analysis, psychological well-being, flourishing at work

## Abstract

**Highlights:**

**Public health relevance—How does this work relate to a public health issue?**

- Seasonal tourism workers represent a large proportion of the precarious workforce, exposed to psychosocial risks including job insecurity, emotional exhaustion, and lack of organizational support, all of which have consequences for mental health and occupational well-being.
- This study addresses a gap in public health research by exploring the subjective experience of well-being in this population through a qualitative approach, highlighting personal and relational resources that may sustain flourishing in temporary employment contexts.

**Public health significance—Why is this work of significance to public health?**

- The findings suggest that flourishing at work is achievable even within structurally precarious conditions, offering a positive psychology perspective on occupational well-being in non-standard employment.
- Trust, relational quality, and perceived justice emerge as key protective factors against psychosocial risks, with implications for how workplaces in the tourism and hospitality sector can be designed to support workers’ psychological well-being.

**Public health implications—What are the key implications or messages for practitioners, policy makers and/or researchers in public health?**

- Organizations in seasonal employment contexts should invest in a positive relational climate, soft skills training, and fair employment practices as concrete strategies for psychosocial risk prevention and the promotion of well-being, even within short-term contracts.
- Policy makers should incorporate well-being and flourishing as legitimate outcome indicators in frameworks addressing precarious work.

**Abstract:**

Seasonal workers in the tourism sector are exposed to significant psychosocial risks, such as work overload, emotional exhaustion, and precarious employment conditions. Despite growing interest in positive organizational psychology, little is known about how organizational culture impacts perceptions and experiences of seasonal workers in Italy. This study explores the role of positive organizational culture in promoting well-being among seasonal workers in the tourism sector, examining their direct perspectives on organizational climate, work challenges, and individual and organizational resources. Eight semi-structured interviews were conducted with seasonal workers employed in the hospitality industry in Italy. Data were analyzed through an integrated mixed-method approach combining Grounded Theory methodology with quantitative lexical analysis using T-LAB software, ensuring both analytical rigor and interpretive depth. Five macro-categories emerged inductively from the data: trust and relations, coping strategies and emotions, perceived justice, teamwork, and meaning of work. These were integrated into a core category defined as flourishing at work, interpreted through the lens of Seligman’s PERMA model. These findings suggest that well-being in seasonal work is an active and relational achievement, sustained by emotional self-regulation, perceived fairness, and collective identity. The results carry direct implications for organizational policies and psychosocial risk prevention strategies in precarious work contexts. In particular, positive organizational culture and environments can act as protective factors against psychosocial risks, with direct implications for organizational policies, psychosocial risk prevention, and evidence-based workplace interventions. The specificity of the analysis method offers an original contribution by integrating qualitative and quantitative textual analysis to investigate psychosocial well-being in an under-explored population: Italian seasonal workers.

## 1. Introduction

The tourism and hospitality industry represents one of the most prominent contexts in which precarious work is situated, considering its reliance on flexible and seasonal arrangements [1]. Seasonal workers face several kinds of demands—physical, emotional, and psychological—and they typically operate under fixed-term contracts of limited duration, are exposed to irregular and unpredictable working hours, and experience limited access to training and career development opportunities [1,2]. Despite these challenges, research has also consistently shown that workers in precarious or seasonal contexts can develop adaptive strategies, derived from relational and organizational resources, and can maintain good levels of engagement and well-being [1,2,3]. In this study, workplace well-being is defined as a multidimensional construct that includes emotional, cognitive, behavioral, personal, and social dimensions of functioning at work, combining hedonic aspects, such as positive affect and job satisfaction, with eudaimonic aspects, such as meaning, autonomy, competence, and self-realization [4,5,6].

A prevalent strand of research has adopted job satisfaction and turnover intention as primary outcome variables, often ignoring broader constructs such as meaning of work, flourishing, and psychological growth at work [7,8]. The Job Demands-Resources model (JD-R) [9] has often been implemented as a key theoretical framework, providing a conceptual baseline for understanding how job demands, such as workload intensity, emotional labor, and precarious contract, interact with job resources, which can include social support, autonomy, and organizational climate, to predict well-being. Studies conducted in tourism contexts have confirmed the relevance of this model, demonstrating that resources such as supervisory support, team cohesion, and a positive organizational climate can buffer the adverse effects of high job demands [2,10]. From a psychological perspective, understanding how workers tackle precarious employment conditions requires attention to both objective work characteristics and their subjective psychological experience; nevertheless, the field remains fragmented and under-explored, particularly with respect to qualitative methods and subjective dimensions of well-being, such as meaning, flourishing, and psychological growth [3,11]. While the JD-R model provides a framework for identifying the structural conditions that sustain or undermine well-being, it does not fully account for the subjective and experiential dimensions through which workers actively construct meaning and flourishing in their work lives. Positive psychology offers a useful lens to analyze these dimensions. In this framework, flourishing can be understood as a multidimensional and dynamic process through which individuals build and sustain personal and collective well-being across different life domains, including work. A key theoretical operationalization of flourishing is represented by Seligman’s PERMA model [12], which identifies five interconnected components—positive emotions, engagement, relationships, meaning, and accomplishment—as the constitutive dimensions through which flourishing is experienced and sustained.

These five elements suggest that well-being at work is not the pure absence of stress, but rather the active and multidimensional process of growth and meaning making [13].

In the present study, this model is adopted as an interpretive framework to situate the findings within an established positive psychology perspective.

Originally conceived at the individual level, the PERMA model has been increasingly applied in organizational and occupational contexts to understand the conditions that foster employee well-being, motivation, and resilience. On the other hand, it is important to know what those conditions are that may destabilize well-being at work: psychosocial risks represent a growing concern in evolving workplaces; indeed, the European Agency for Safety and Health at Work (EU-OSHA) identified psychosocial risks as a priority concern in evolving workplace environments [14]. These risks include high job demands, low control or autonomy, lack of social support, role ambiguity, job insecurity, and emotional exhaustion, all of which are particularly salient in seasonal and temporary employment contexts [1,15].

Despite this awareness, most existing literature has adopted quantitative designs, without deeply exploring the subjective and personal workers’ perspectives [3,16]. Thus, the present study addresses this gap by adopting a qualitative approach, focusing on workers’ personal experiences; qualitative methods are particularly suited to capture the inner nature of well-being experiences, and the mixed-method analysis can enhance analytical rigor and interpretative depth [17].

The aim of the study is to explore the personal, contextual, or relational resources that seasonal tourism workers develop to cope with psychosocial risks typical of temporary employment. More specifically, the study addresses the following research question: how do seasonal tourism workers in Italy perceive, narrate, and make sense of these resources within their daily work experience?

## 2. Materials and Methods

Data were collected through in-depth semi-structured interviews; this qualitative methodology was adopted to elicit a vivid picture of the participants’ perspective and explore their personal perceptions.

The study, qualitative and non-interventional in nature, was conducted in accordance with the ethical principles applicable to research involving human subjects, with risk minimization, protection of confidentiality, and acquisition of written informed consent from participants. All research procedures complied with international ethical standards and with the principles outlined in the Declaration of Helsinki [18]. Moreover, anonymity was granted in accordance with EU Regulation 2016/679 [19] and participants were informed about the handling of personal data.

Methodological quality was supported by the application of the standard for reporting qualitative research (SRQR) [20] and of the Critical Appraisal Skills Program [21], thus ensuring methodological rigor in the research design.

The interview was created ad hoc according to the explorative aim of the study; some questions are reported as follows:

- What is the most challenging moment of the summer season and why?
- What is the most important value within the company you work for?

During the interview, the interviewer used probing questions to clarify unclear answers, and also asked participants to give examples to support their narrations.

### 2.1. Sample

Eight employees working in the tourism and hospitality sector in Southern Italy were recruited through a purposive sampling. They were based in two towns in the same region, both widely recognized as top summer tourism destinations. The sample covered three distinct organizational settings: four restaurants, three hotels, and one tourist market. Seven participants held front-line employee roles; one participant, employed in the restaurant sector, held a managerial position. The sample comprised seven males and one female, with a mean age of 44.4 years (range: 25–65) and an average of 17.1 years of experience in the field (sample descriptives in Table 1).

Although the sample is intentionally small, this is consistent with the explorative orientation of the study, in which analytical depth and the richness of individuals’ contributions are more relevant over quantity. Data collection continued until thematic saturation was reached, with no new substantive themes emerging after the sixth interview. Saturation was assessed through a systematic comparison of emerging themes across successive interviews: after each interview, new data were checked against the existing thematic map to identify any novel patterns. From the sixth interview onwards, no new codes or themes were generated, confirming that saturation had been reached. The geographic and sectoral homogeneity of the sample, while limiting generalizability, strengthens the internal coherence of the findings and permits possible comparisons with similar market context.

### 2.2. Mixed-Method Analysis

The present study adopts a qualitatively dominant embedded mixed-method design [22,23], in which a primary qualitative strand—thematic analysis—is complemented by a quantitative component—software-assisted discourse analysis—applied to the same interview corpus. This design reflects an asymmetric, yet genuinely integrative, methodological architecture: the two components are analytically distinct, address the research questions from different vantage points, and are brought together at the interpretive stage through methodological triangulation.

This method was chosen to guarantee a balance between the explorative and heuristic dimensions of qualitative inquiry and the systematicity of structured textual examination. As Cortini and Tria [17] demonstrate, software-assisted lexical and co-occurrence analysis of interview transcripts operates at the intersection of qualitative and quantitative epistemologies, generating a distributionally grounded layer of evidence that complements, without replacing, interpretive thematic coding. The integration of these two methods allowed for methodological triangulation, enhancing the trustworthiness and credibility of the findings in accordance with SRQR guidelines [20] and the guidelines for qualitative research [24]. Each interview lasted over 60 min (range: 60—90 min) and was conducted face-to-face in a neutral location, ensuring a comfortable and naturalistic setting for disclosure; interviews were audio-recorded with the participants’ consent and subsequently transcribed verbatim.

The interview guide was developed based on a review of the existing literature on the topic (it is available as Supplementary Materials). Key thematic areas were identified from prior research and translated into open-ended questions designed to encourage participants to elaborate freely on their experiences. This approach ensured both theoretical grounding and sufficient flexibility to capture unanticipated themes.

Researchers were external to the tourism and hospitality sector and had no prior professional or personal ties to the regional context under investigation. This outsider positioning is considered a methodological asset, as it reduced the risk of assumption-driven interpretation and supported an open analytical attitude.

Thematic analysis constituted the primary analytical strand and followed Corbin and Strauss’ [25] bottom-up approach, articulated in three coding steps: with the open coding, two researchers independently attributed codes to significant words and sentences, and compared, reviewed and discussed their coding. Researchers kept an open mindset in order to be sensitive to those concepts or themes emerging naturally from raw data.

Axial coding: Looking for relationships between codes, they were collected into larger code groups and then framed into wider macro-categories. Codes sharing semantic or conceptual affinity were progressively clustered into sub-categories. This grouping phase emerged iteratively through constant comparison across interviews: researchers examined how codes related to one another, identified recurring patterns, and discussed boundary cases until agreement was reached on the composition of each sub-category. Sub-categories were then aggregated into broader macro-categories based on their conceptual coherence and thematic relevance to the research questions.

Selective coding: In this final phase, researchers were looking for a theoretical explanation supported by the data and they identified a core category capable of integrating and theoretically connecting all other categories. The core category was selected because of its centrality, its frequency across the data, and its explanatory power in relation to the aim of the study.

To interpret and theoretically situate the core category, the PERMA model [12] was adopted as an interpretive lens. This framework was not introduced a priori to guide data collection or early coding phases but was applied in the final interpretive stage to examine whether and how the inductively derived macro-categories could be mapped onto an established positive psychology perspective. The choice of PERMA was informed by its multidimensional structure and its established applicability to occupational well-being research [26,27].

Each sentence, paragraph, or passage representing an idea named by a participant was considered a unit of meaning. The smallest unit of meaning considered was a sentence containing at least one verb and one subject. Two researchers trained in qualitative analysis coded the interviews to identify and categorize the participants’ discourse, with a subsequent inter-rater agreement calculated thanks to Cohen’s Kappa (0.85). Then, a third researcher coded a subsample of the full interviews to resolve ambiguities in code definitions and refine the segmentation criteria.

Discourse analysis constituted the supplementary quantitative strand and was conducted using T-LAB 10.2 software, which enabled the quantitative examinations of the transcripts through the identification of significant word repetitions and exploration of frequent associations and co-occurrences within the text. This approach is specifically suited to the analysis of language and textual data, including interview transcripts [17], and complemented the qualitative coding by revealing structural patterns in participants’ discourse that were not directly accessible through interpretive coding alone.

Thematic analysis was selected as the primary method because the study aims to provide a descriptive and interpretive account of participants’ experiences, and it is well-suited for identifying and analyzing themes and patterns within qualitative data [28].

The discourse analysis findings were integrated with the thematic results at the interpretative stage, with points of convergence and divergence between the two analytical layers discussed in relation to the research questions. This mode of integration—occurring at the level of interpretation rather than data collection—is characteristic of the embedded mixed-method design [23], and reflects the asymmetric but complementary role of the two components in the overall analytical architecture [29].

## 3. Results

The iterative process of analysis and interpretation resulted in 241 codes that were grouped into five macro-categories, which emerged inductively from the data through the progression from open to axial coding. The categorization was considered complete when theoretical saturation was reached; thus, the macro-categories referred to specific dimensions of participants’ experience, and include trust and relations; coping strategies and emotions; perceived justice; teamwork; and meaning of work (Table 2). Theoretical saturation of the macro-categories was determined by verifying that no new codes generated during later stages of analysis required the creation of additional categories, and that each emerging code could be consistently included within the existing categories. Transcripts were reviewed iteratively to identify any deviant data.

### 3.1. Trust and Relations

This macro-category collects codes related to trust, mentioned as a core value and as a concrete practice as well, and to relationships. It seems that trust is described as the main prerequisite for different levels of work life, such as manager–employee relation, colleagues’ reciprocity, and customers’ reciprocal recognition: “they come back years later and still recognize you” (P.2); “trust and collaboration are the real foundation” (P.1).

Trust is the most frequently quoted value across participants. It is not described as a mere abstract value; rather, trust is experienced as a real practice, which is co-constructed together with other internal or external organizational entities.

### 3.2. Coping Strategies and Emotions

In this macro-category there are codes related to personal resources and emotions. Participants make several references to the personal soft skills they implement when facing peak season fatigue. One worker said: “I’m happy when I feel adrenaline, but also afraid” (P.3).

Other examples are: “I motivate myself; I tell myself to do better”, or “my secret powers are calm, tranquility and a smile” (P.5).

Emotional self-regulation is also evident by other participants who stated: “I think of my family, when I know I have to stay calm” (P.8), or “I try to see the positive in everything” (P.1).

### 3.3. Perceived Justice

Codes detected in this macro-category are those concerning perceptions about how the organization cares for employees. Economic recognition is a shared signal of support: “I had an economic difficulty and he [the manager] helped me” (P.2), and “I can’t complain, there is a good salary” (P.4). There are also references to the advantage of being in the condition of dignified work—someone recalled an earlier experience where they were “treated like slaves, as at the time of ancient Egypt” (P.6), whereas in their current occupation: “I feel considered as a person, not exploited” (P.8).

### 3.4. Teamwork

Within this macro-category there are codes related to positive team spirit and to the collective dimension as a family; there is also a negative dimension concerning new staff or generational tension.

Experiences include workers mentioning that “We are in full harmony” (P.2), or “we help each other in the kitchen” (P.4), or “it has been created a real team game” (P.6) as positive evidence of a collective identity.

The family metaphor has also been used to normalize conflict—“in every family there are arguments” (P.3)—or as a positive value: “the workplace must be a big family” (P.5).

On the negative side, a participant made complaints about newcomers who were demonstrating low work ethic: “they come just to get through the day” (P.7). A different problem also seems to be knowledge transfer: “you say A and they do B, a lot of patience is needed…” (P.6). There is also an employee who is worried about the shortage of qualified staff: “you can’t find people who want to do this job” (P.1).

### 3.5. Meaning of Work

Codes related to the meaning of work are represented by personal expertise and by human contact meant as an intrinsic reward. For example, participant 3 stated that “I’ve been doing this since I was 14”—in this case, the experience is presented as a coping resource.

Further experiences include participants mentioning that “Lots of people come, and that’s what I really like” (P.2), or “Sometimes it becomes a friendship” (P.5): here, employees show how interactions with customers are experienced as a joy, making an explicit reference to one of their inner meanings of work.

### 3.6. Quantitative Evidence: T-LAB

The corpus derived from the interviews was also analyzed with a quantitative approach, with the support of the statistical software T-LAB, which can provide a visual map of the content generated by participants. Before running the analyses, the text was prepared with a detailed lemmatization; thus, words with the same root meaning are clustered together (such as work and working). Authors at first carried out an automatic analysis of the content to see the word occurrences. This first step has an explorative aim: the most cited words or themes are those most relevant to participants.

The second kind of exploration was through co-occurrences: T-LAB software delivers a graphical output that shows the target word in the middle, with all the others around—the closer words are those that co-occur the most with the target.

T-LAB allows the user to create customized representations as well; for example, to put a specific word of interest in the middle to create a graphical representation of its associations.

**Figure 1:**
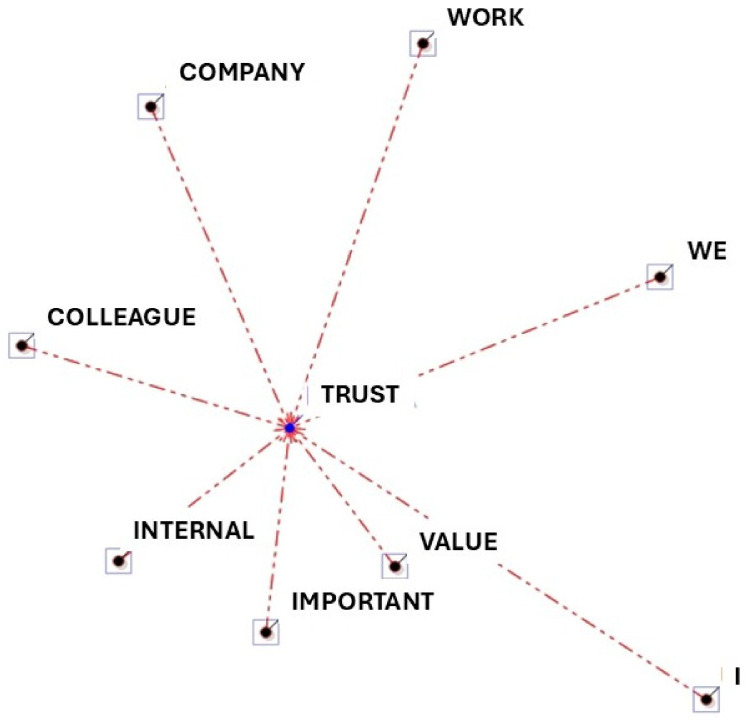
Co-occurrences with the lemma TRUST.

Concerning our study, the first automatic exploration returned “trust” as the most frequently occurring word in the corpus (29), along with its significant co-occurrences, confirming the definition of the macro-category “trust and relations” through the thematic analysis. Figure 1 shows the graphical representation and Table 3 reports the coefficient of cosine with the *p* significance.

Observing the co-occurrences, the lemma trust has the most significant correlations with value (cosine coefficient: 0.60) and with important (cosine: 0.53), suggesting that participants conceptualize trust as a core value related to professional contexts, and the attribute “important” may confirm that trust is entangled in their work experience. The co-occurrence with the word internal (cosine: 0.46), also in relation to the macro-category “teamwork”, suggests that trust is intrinsically connected to internal organizational dynamics. Moreover, it is worth noting the connection with the dyadic nature of trust through the word “colleague” (cosine: 0.34), and at the organizational level through the word “company” (cosine: 0.30).

**Figure 2:**
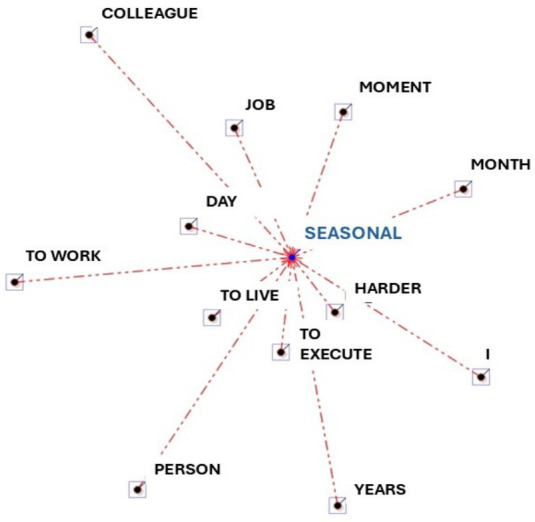
Co-occurrences with the lemma SEASONAL.

**Figure 3:**
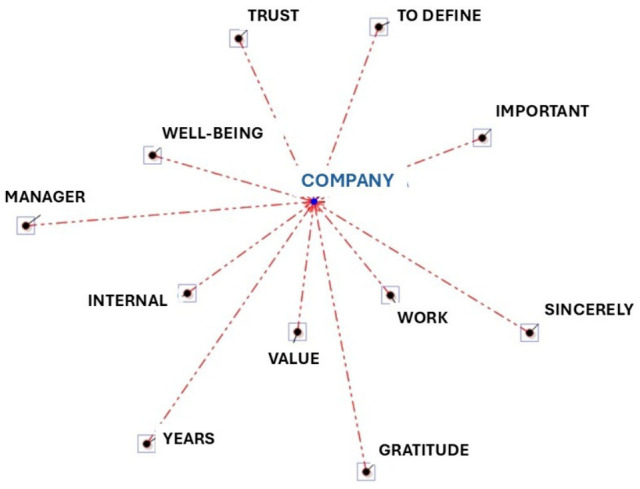
Co-occurrences with the lemma COMPANY.

Researchers decided to run a personalized exploration asking the software to elaborate the co-occurrences for the words “seasonal” (Figure 2) and “company” (Figure 3). The choice to analyze the word “seasonal” was theoretically driven by the nature of the sample itself; as they were seasonal workers, it was relevant to capture how participants shaped this temporary employment condition. The decision to investigate the word “company” was motivated by the findings of the initial automatic analysis, which identified it as a significant co-occurrence with “trust”. Also, the qualitative thematic analysis depicted the organizational dimension as a resource for the mitigation of psychological risks related to the typical pressure of seasonal jobs.

Experiential and temporal dimensions are the main semantic field described in relation with the lemma “seasonal”; for example, as showed in Table 4, the word “harder” (cosine: 0.59) indicates that participants explicitly perceive their work experience as particularly demanding.

The co-occurrence with the verb “to execute” (cosine: 0.45) may suggest that the focus of workers is on practical and operational demands. Notably, when the performing focus is framed with the co-occurring verb “to live” (cosine: 0.39), which makes the experience of seasonal work actively lived, it echoes the concept of engagement and presence. Words like “day”, “moment”, and “month”, even if they do not all reach statistical significance, reinforce that participants are anchored to routines and the temporal dimension.

Finally, Figure 3 and Table 5 represent the co-occurrences and cosine coefficients of the word “company”.

The strongest association with the lemma “company” is the word “work” (cosine 0.44), confirming participants’ operational commitment to their workplace. Notably, however, other semantically significant co-occurrences emerge that are theoretically central to the positive psychology framework: the words “value” (cosine: 0.42) and “trust” (cosine: 0.31) suggest that participants do not perceive their organization merely as a functional context, but as a relational and value-laden environment.

The co-occurrence with “well-being” (cosine: 0.31) may suggest that the organization is perceived as a positive space and as a source of psychological flourishing, thus representing a protective factor against psychosocial risk.

Overall, the T-LAB analysis revealed that participants’ discourse was predominantly organized around three key concepts: the organization, the summer season, and trust, which together reflect in part the core category “flourishing at work”. The company provides a meaningful and trust-based structural setting, the summer season represents a particularly demanding but also emotionally dense period, and trust emerges as the relational foundation that makes flourishing possible.

## 4. Discussion

The present study aimed to explore how seasonal tourism workers experience well-being at work and which personal, relational, and contextual resources sustain their capacity to flourish despite the psychosocial risks associated with temporary employment. Through a mixed-method analysis combining grounded theory and lexical analysis, the findings revealed a coherent picture in which relationships and positive organizational values function as protective factors against typical psychosocial risks of seasonal work. The integration of five inductively derived macro-categories yielded a core category—flourishing at work—understood as a multidimensional and multicomponent process through which workers build well-being even within the structural constraints of precarious and seasonal employment.

The interpretation of this core category was guided by Seligman’s PERMA model [12], which provided a theoretical scaffold to situate the inductively derived categories within an established positive psychology perspective. This approach differs from previous applications of PERMA in tourism research, which have predominantly relied on survey-based, top-down measurement strategies [30,31]. The present study, by contrast, demonstrates that PERMA dimensions find a bottom-up correspondence in participants’ lived experiences, suggesting that flourishing is not merely an outcome to be measured but a process actively constructed by workers themselves. Each macro-category engages with the existing literature in distinct ways.

Regarding trust and relations, they correspond to the “relationships” dimension of PERMA. According to the results, trust is not described by participants as a declared criterion but as a concrete and co-constructed practice, built and experienced through daily interaction with managers, colleagues and customers alike. This finding resonates with Edmondson’s conceptualization of psychological safety and interpersonal trust as distinct but complementary constructs [32]. While trust lowers the costs of social interaction and reduces the need for monitoring, psychological safety creates the conditions for open communication and learning. In the context of seasonal work, where the time frame for relationship building is limited, the salience of trust in participants’ accounts suggests that relational quality may compensate for structural instability, functioning as a psychological resource that buffers the adverse effects of precarious employment.

Regarding coping strategies and emotions, participants drew on a repertoire of emotional self-regulation strategies—reframing, self-motivation, cultivating calm—to cope with the physical and psychological demands of peak season. Indeed, participants referred to soft skills such as calm, patience and other positive emotions as their main resource to face challenges and raise engagement. These emotional competencies align with the “positive emotions” dimension of PERMA and reflect what Fredrickson’s broaden-and-building theory [33] describes as the capacity of positive emotions to build durable personal resources over time. In the hospitality industry, intrinsic motivation has been shown as a coping strategy for role conflict and negative emotions, enhancing frontline employees’ capacity to sustain engagement under high-demand conditions [34]. Our findings extend this evidence to the seasonal work context, suggesting that emotional self-regulation is not merely a coping mechanism but a generative resource that workers proactively cultivate. With respect to perceived justice, connected to the “engagement” dimension of PERMA, participants’ references to economic recognition and relational dignity—being treated “as a person, not exploited”—suggest that fairness is experienced not merely as formal entitlement but as a relational signal that activates psychological investment in work. This is consistent with social exchange theory [35,36], which posits that employees who perceive organizational support and fair treatment feel obligated to reciprocate through increased loyalty and engagement. Saks [37] demonstrated empirically that this reciprocity mechanism is a key antecedent of work engagement; when individuals receive socio-emotional resources from their organization, they repay through deeper cognitive, emotional, and physical investment in their roles. The contrast evoked by participants between past exploitation and current dignified treatment further suggests that justice is experienced as a continuum whose positive pole directly sustains psychological presence at work.

Teamwork connects again to “relationships” as a PERMA dimension, this time at the collective level. The family metaphor recurrently used by participants to describe their workplace relationships indicates a form of affective bonding that goes beyond functional cooperation. This is consistent with Social Identity Theory [38], which explains how identification with a group generates a sense of belonging, shared purpose, and prosocial behavior. Research on group cohesiveness further confirms that members of cohesive groups—who often describe themselves using terms such as “family” or “community”—experience less anxiety, cope better with stress, and perform more effectively under high-demand conditions [39,40]. In the seasonal work context, where teams form rapidly and dissolve at the end of each season, the emergence of this collective identity represents a particularly significant psychological achievement, acting as a shared resource capable of absorbing tension and normalizing conflict, while also generating a sense of purpose and solidarity.

Finally, regarding meaning of work, participants located the meaning of their work primarily in the intrinsic reward derived from customer interactions and in the personal expertise accumulated over years of seasonal employment. This bottom-up process of sense-making aligns with the “meaning” and “accomplishment” dimensions of PERMA and resonates with research on intrinsic motivation in hospitality settings, which shows that employees who find intrinsic satisfaction in relational and service-oriented aspects of their work display higher engagement and resilience [34]. Importantly, participants’ accounts suggest that meaning is not organizationally assigned but individually and relationally constructed—a finding that extends the existing literature by highlighting workers’ agency in the meaning-making process even within structurally constrained employment contexts.

Taken together, these five macro-categories converge into the core category of flourishing at work, suggesting that even in the context of seasonal and precarious employment, there are possibilities to develop and sustain a rich and multidimensional experience of well-being, as the participants in this study demonstrated.

Notably, this process appears to be neither passive nor incidental: it emerges from the active cultivation of relationships, emotions, and meaning, suggesting that flourishing in precarious work contexts is less a structural given than a relational and individual achievement. These findings align with and extend Verma’s [41] integrative review of flourishing at work, which identifies relational and cognitive resources as key antecedents of workplace flourishing, while also noting that precarious work conditions reduce workers’ capability sets. Our study complements this picture by providing qualitative, bottom-up evidence that flourishing is possible even in precarious contexts, and that it is sustained by workers’ proactive agency rather than by structural conditions alone. This represents a contribution to the emerging positive psychology of precarious work, which—as Benach et al. [42] argued—has too often focused exclusively on the negative mechanisms linking precarity to ill-being, and neglecting the psychological resources through which workers construct resilience and meaning.

However, the study has several limitations related to its qualitative and explorative nature. First, the small sample size (N = 8) and cross-sectional design constrain statistical power, representativeness, and the ability to observe temporal or seasonal variations in worker experiences. Second, potential selection bias may have systematically favored more engaged or articulate participants who volunteered for interviews, thereby excluding voices of workers with more negative or withdrawn responses. Third, social desirability effects could have influenced participants’ responses, as interviewees might have emphasized positive aspects of their work experience (e.g., teamwork, relationship) while downplaying challenges or dissatisfaction. Fourth, the limited diversity of organizational contexts—data was collected from a narrow range of firms and geographic regions—restricts the applicability of findings across the varied tourism landscape. The sample is also limited in terms of demographic diversity (origin, gender, occupational role). Future research would benefit from larger, more geographically and organizationally diverse samples, alongside mixed-methods approaches that integrate qualitative insights with validated quantitative measures of PERMA dimensions and psychosocial risks.

Notwithstanding these limitations, the study contributes to existing literature from both a theoretical perspective and a practical level of intervention.

From a theoretical standpoint, the primary contribution is the application of the PERMA model to the seasonal tourism sector through an exclusively bottom-up, inductive approach. Previous applications of PERMA in tourism research have predominantly implemented survey, top-down measurement approaches [30,31]; the present study demonstrates that each of the five PERMA dimensions finds a correspondence in the experience of seasonal workers, suggesting that the model captures dimensions of well-being that are not only measurable but organically experienced.

Second, specific themes raised by participants—such as trust, relationship, meaning of work, and perceived justice—are described not as obvious countermeasures against job demands, but rather as dimensions through which workers in this sample experience and articulate well-being. In their accounts, participants described activating relational and cognitive resources to sustain their well-being, suggesting that workers may develop proactive strategies even within challenging conditions. These preliminary observations warrant further investigation in larger samples. Third, workers described experiences of meaning, connection, and growth despite the constraints of precarious employment. While these findings are theoretically interesting and suggest that positive psychological experiences are possible in such contexts, they represent insights from a small and specific sample and need to be validated in broader and more diverse research contexts. Finally, findings suggest trust as a fundamental variable to study for well-being in seasonal and temporary workers; it was one of the most salient themes raised by participants, meant as a workplace value but also as something that is actively co-constructed through reciprocal interactions, becoming one of the protective factors that can alleviate psychosocial risks inherent to temporary employment.

From a methodological perspective, the combination of grounded theory and T-LAB lexical analysis represents an original analytical strategy in this field, allowing interpretive depth to be systematically supplemented by corpus-based verification of semantic patterns, thereby enhancing the credibility and transparency of qualitative findings.

Regarding the practical implications of the study, findings from this sample suggest that relational climate may be an important factor in supporting worker well-being, a consideration that becomes especially relevant given the limited timeframe of seasonal employment. Investment in soft skills training remains valuable even for workers on short-term contracts, as it may strengthen their capacity to cope with the psychosocial demands of seasonal work. Innovative approaches such as gamification have also shown potential in enhancing self-efficacy and positive attitudes toward work-related challenges [43]. At a broader policy level, well-being and flourishing may merit consideration alongside traditional economic indicators when designing regulatory frameworks for precarious work. These observations are exploratory and context-specific; larger, more geographically and organizationally diverse studies will be needed before drawing generalizable conclusions or formulating broader policy recommendations.

Finally, this study identifies several resources that could be better cultivated in the tourism sector; nurturing relationships, trust, and meaning of work may contribute to occupational identity and strengthen commitment and well-being.

Another relevant topic is the experience of teamwork, which participants described using the metaphor of a family, thus highlighting the desire and importance of belongingness and framing the group as a shared psychological resource [44]. More broadly, participants’ accounts suggest that flourishing can emerge through relational, cognitive and emotional resources, even in precarious work contexts. These findings offer preliminary theoretical insight and suggest promising directions for future research.

Findings suggest that flourishing is not absent in precarious or seasonal work, but is negotiated through relational, cognitive, and emotional resources. Organizations operating in the tourism sector might consider investing in trust-building practices, fair management, and team cohesion that emerged prominently from participants’ experiences as preventive strategies against psychosocial risks, rather than focusing exclusively on individual-level interventions. These exploratory insights suggest promising directions for future investigation in diverse organizational contexts.

## 5. Conclusions

The present study aimed to investigate the subjective experience of well-being among seasonal tourism workers, with particular attention to the personal, relational, and contextual resources that sustain flourishing in the face of psychosocial risks inherent to temporary employment. Through a qualitative bottom-up approach, the study generated a theoretically coherent picture in which flourishing at work emerges not as an exceptional outcome, but as an active, co-constructed process, characterized by trust, meaningful relationships, coping strategies, perceived justice, teamwork, and a sense of purpose derived from work itself.

The convergence of five macro-categories into a single core category, interpreted through the PERMA framework, suggests that positive psychology offers a productive and underutilized lens for understanding well-being in seasonal employment contexts. At the same time, the findings suggest that flourishing in these contexts is not purely individual: it is instead relational, organizational, and in part structural, intertwined with the fairness of employment conditions and the degree to which organizations can actively nurture the conditions for trust and meaning, even within compressed seasonal timeframes.

In the context of Italian seasonal tourism, this study identifies resources such as trust, teamwork, intrinsic meaning and emotional competence, that appear relevant to worker well-being. While these findings emerge from a specific sample, they may suggest directions for investigating well-being in other precarious employment contexts. As precarious and temporary forms of work persist, understanding the conditions that enable worker well-being becomes a theoretical and practical priority. However, whether these dynamics operate similarly across different sectors, geographies, and employment arrangements remains an open empirical question requiring future research in larger and more diverse samples [45].

Ultimately, this study invites a reframing of seasonal tourism workers not as a merely vulnerable population defined by structural precariousness, but as individuals who, in this sample, described experiences of agency in constructing meaning, trust, and well-being even within the constraints of temporary employment. Whether these capacities generalize across different contexts and populations remains an important question for future research, one that may contribute to dialog between positive psychology, occupational well-being research, and policy frameworks addressing psychosocial risk prevention in non-standard and evolving workplaces.

## Figures and Tables

**Table 1:** Sample descriptives.

Participant	Gender	Age	Job Role	Years of Experience
1	F	62	Restaurant staff	10
2	M	65	Restaurant manager	35
3	M	37	Restaurant staff	10
4	M	26	Restaurant staff	5
5	M	60	Hotel receptionist	30
6	M	47	Hotel receptionist	30
7	M	25	Hotel receptionist	2
8	M	33	Retail sales assistant	15

**Table 2:** Coding process.

Codes	Categories	Macro-Categories
Trust; relationships	Interpersonal trust	Trust and relations
Personal resources; emotions	Emotional and personal resources	Coping strategies and emotions
Care; economic recognition; decent work	Organizational fairness	Perceived justice
Positive spirit; family dimension; generational tensions	Organizational climate and culture	Teamwork
Expertise; human relations	Professional and relational competencies	Meaning of work

**Table 3:** Coefficient of cosine and chi2 of co-occurrence with the lemma Trust.

LEMMA_B	COEFF	CE_B	CE_AB	CHI2	(*p*)
Value	0.6	10	9	91.65	<0.001
Important	0.53	26	13	66.1	<0.001
Internal	0.46	10	7	52.29	<0.001
Colleague	0.34	30	9	21.03	<0.001
Company	0.3	56	11	12.03	0.001
Work	0.26	139	15	2.38	0.123
We	0.24	12	4	10.43	0.001
I	0.2	40	6	2.82	0.093

**Table 4:** Coefficient of cosine and chi2 of co-occurrence with the lemma SEASONAL.

LEMMA_B	COEFF	CE_B	CE_AB	CHI2	(*p*)
Harder	0.59	10	10	89.41	<0.001
To execute	0.45	11	8	47.77	<0.001
To live	0.39	14	8	34.58	<0.001
Day	0.36	17	8	26.12	<0.001
Job	0.3	139	19	3.18	0.074
Moment	0.28	45	10	8.06	0.005
Month	0.21	7	3	8.12	0.004
I	0.18	40	6	1.06	0.302
Years	0.17	19	4	2.48	0.115
Person	0.15	25	4	0.92	0.336
To work	0.14	29	4	0.40	0.526
Colleague	0.14	30	4	0.31	0.577

**Table 5:** Coefficient of cosine and chi2 of co-occurrence with the lemma COMPANY.

LEMMA_B	COEFF	CE_B	CE_AB	CHI2	(*p*)
Work	0.44	139	39	11.01	0.001
Value	0.42	10	10	41.30	<0.001
Internal	0.34	10	8	23.22	<0.001
Well-being	0.31	12	8	16.97	<0.001
Trust	0.31	23	11	12.04	0.001
To define	0.29	10	7	16.12	<0.001
Important	0.28	26	11	8.83	0.003
Sincerely	0.20	15	6	3.92	0.048
Gratitude	0.20	11	5	4.60	0.032
Years	0.18	19	6	1.68	0.195
Manager	0.18	9	4	3.44	0.063

## Data Availability

Data presented in this study are available on request from the corresponding author due to privacy and ethical restrictions related to the qualitative nature of the material and the confidentiality of interviews.

## Supplementary Materials

The following supporting information can be downloaded at: https://www.mdpi.com/article/10.3390/ijerph23060779/s1, File S1. Interview Guide.
