# Supplementary material for: From Risk to Flourishing: Organizational Resources in Seasonal Tourism Work
Source: Int J Environ Res Public Health. 2026 Jun 10;23(6):779. doi: 10.3390/ijerph23060779 (PMC13300468; doi:10.3390/ijerph23060779)
Supplement: Supplementary file 1 [file ijerph-23-00779-s001.zip › ijerph-4266325-supplementary.pdf]

First of all, thank you for your time. Could you tell me about your work history at the company you currently work for?

How does your working day unfold during the summer season?

What do you enjoy about this job?

What are the main challenges you face in your daily work?

What is the most difficult moment of the summer season, and how do you experience it? Could you tell me about a specific episode and how you dealt with it?

In your opinion, what are your personal resources for coping with difficult moments?

In your opinion, what is the most important value within the company you work for?

Does the company you work for take care of your wellbeing? In what way?

What would you improve in the way work is organised?

To conclude, I would like to ask you for three words to describe the company you work for.
